# Supplementary material for: Phenylalanine-tRNA aminoacylation is compromised by ALS/FTD-associated C9orf72 C4G2 repeat RNA
Source: Nat Commun. 2023 Sep 16;14:5764. doi: 10.1038/s41467-023-41511-3 (PMC10505166; doi:10.1038/s41467-023-41511-3)
Supplement: Supplementary file 1 — Supplementary Information [file 41467_2023_41511_MOESM1_ESM.pdf]

SUPPLEMENTARY FIGURES AND TABLES

Phenylalanine-tRNA aminoacylation is compromised by ALS/FTD-associated C9orf72 C4G2 repeat RNA

Mirjana Malnar Črnigoj[1,2], Urša Čerček[1,2], Xiaoke Yin[3], Manh Tin Ho[4], Barbka Repic Lampret[5], Manuela Neumann[6,7], Andreas Hermann[8,9], Guy Rouleau[10,11], Beat Suter[4], Manuel Mayr[3], Boris Rogelj[1,12*]

[1]Department of Biotechnology, Jožef Stefan Institute, Ljubljana, 1000, Slovenia
[2]Graduate School of Biomedicine, Faculty of Medicine, University of Ljubljana, Ljubljana, 1000, Slovenia
[3]King's BHF Centre, King's College London, London, SE5 9NU, UK
[4]Institute of Cell Biology, University of Bern, Bern, 3012, Switzerland.
[5]Clinical Institute of Special Laboratory Diagnostics, University Children's Hospital, University Medical Centre Ljubljana, Ljubljana, 1000, Slovenia
[6]Molecular Neuropathology of Neurodegenerative Diseases, German Center for Neurodegenerative Diseases, Tübingen, 72076, Germany
[7]Department of Neuropathology, University Hospital of Tübingen, Tübingen, 72076, Germany
[8]Translational Neurodegeneration Section "Albrecht-Kossel", Department of Neurology and Center for Transdisciplinary Neurosciences Rostock (CTNR), University Medical Center Rostock, University of Rostock, 18147, Germany
[9]Deutsches Zentrum für Neurodegenerative Erkrankungen (DZNE), Rostock/Greifswald, 18147 Rostock, Germany
[10]Department of Human Genetics, McGill University, Montréal, QC, H3A 0G4, Canada.
[11]Department of Neurology and Neurosurgery, Montreal Neurological Institute, McGill University, Montréal, QC, H3A 0G4, Canada.
[12]Faculty of Chemistry and Chemical Technology, University of Ljubljana, Ljubljana, 1000, Slovenia

These authors contributed equally: Mirjana Malnar Črnigoj, Urša Čerček

***** To whom correspondence should be addressed: Prof. Boris Rogelj; Email: boris.rogelj@ijs.si;Tel: +386 1 477 34 11; Department of Biotechnology**,** Jožef Stefan Institute, Jamova cesta 39, 1000 Ljubljana, Slovenia

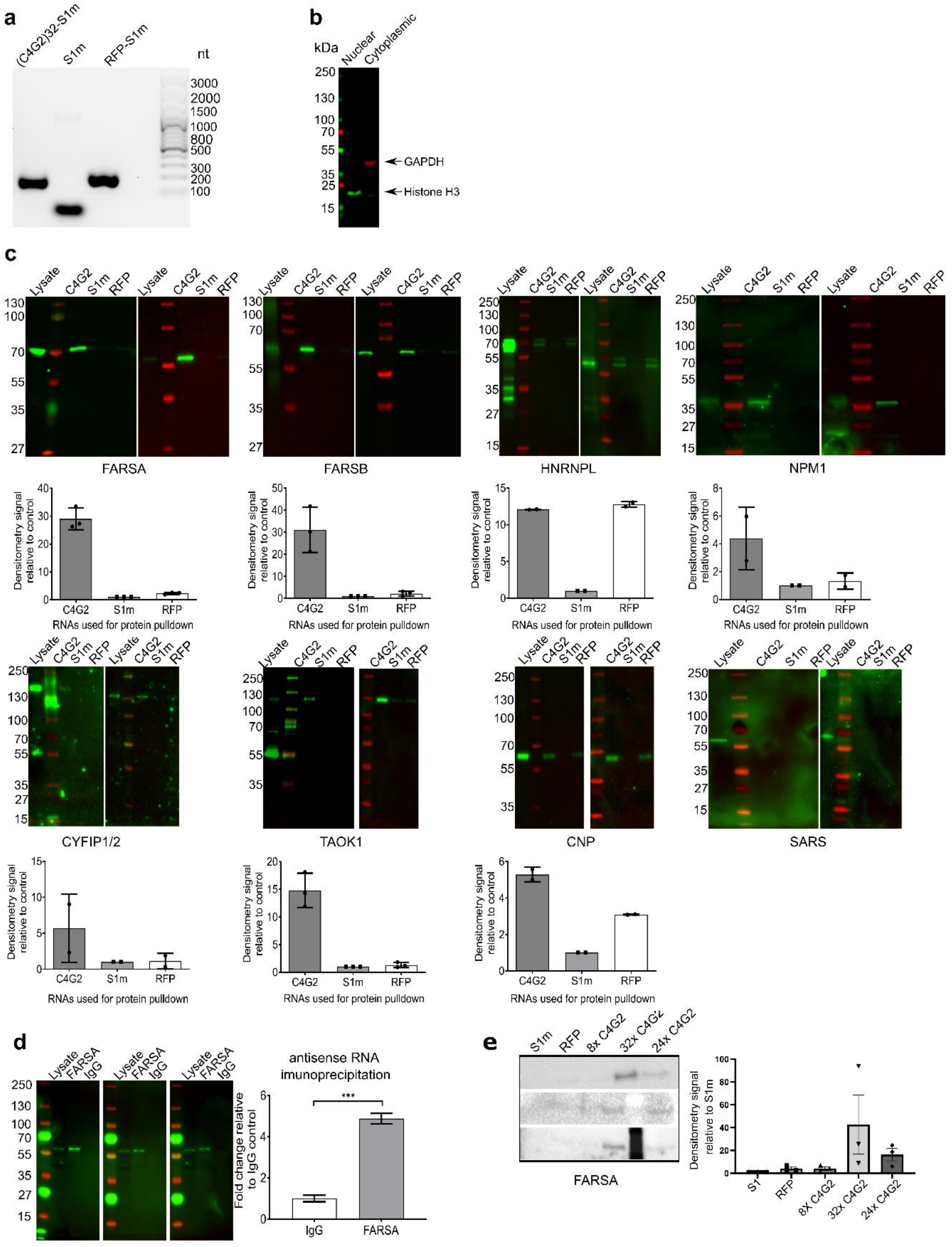

Supplementary Fig. 1 C4G2 RNA constructs bind various proteins in RNA pull-down assays. a) Purity and integrity of RNA constructs used for RNA pull-down assays were confirmed with agarose gel electrophoresis. The RNA constructs were as follows: 32 C4G2 RNA repeats with S1m aptamer on the 3' end, S1m aptamer, and partial sequence of RFP with S1m aptamer on the 3' end. b) Nuclear and cytoplasmic fractions of mouse brain lysates used for RNA pull-down assays were evaluated with

detection of the cytoplasmic protein GAPDH and the nuclear protein Histone H3. c) Western blots of eluates from RNA pull-down assays performed on protein lysates from human postmortem brain tissue confirmed the following protein interactors: Phe-tRNA synthetase subunits A and B (FARSA, FARSB), heterogeneous nuclear ribonucleoprotein L (HNRNPL), cytoplasmic FMR1-interacting protein 1/2 (CYFIP1/2), thousand and one amino acid kinase (TAOK1), 2'3'-cyclic nucleotide 3'-phosphodiesterase (CNP), and nucleophosmin 1 (NPM1). The interaction with Ser-tRNA synthetase (SARS) could not be confirmed. For each protein two side by side western blot membranes are presented from two replicates of RNA pull-down assay. d) Three independent repeats of RNA immunoprecipitation assays were performed for the antisense repeat RNA interaction with FARSA. Normal rabbit IgG antibody was used as a control in immunoprecipitation reactions. Binding of FARSA to the magnetic beads was evaluated with WB. Antisense repeat RNA was detected with qPCR. e) FARSA strongly binds 32x and to lesser extent 24x C4G2 RNA repeats. Binding to 8xC4G2 was barely detectable in three independent RNA pull-down assays. Graphs present mean values ± s.e.m with statistical significance labeled as: ** $p<0.01$. Source data are provided as a Source data file.

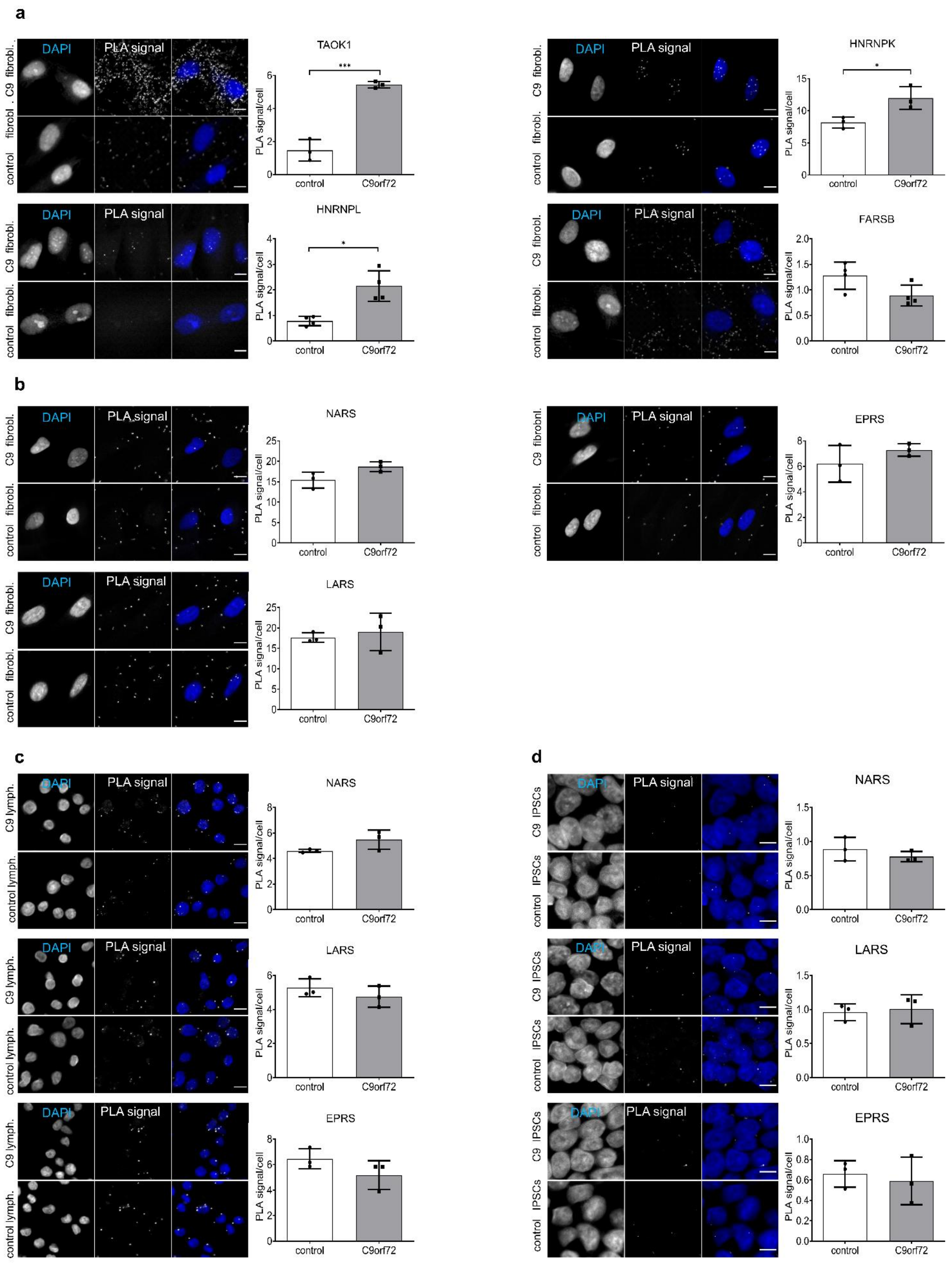

a
b
c
d
DAPI
PLA signal
C9 fibrobl.
control fibrobl.
C9 fibrobnl.
C9 lymph.
control lymph.
C9 IPSCs
control IPSCs
TAOK1
HNRNPK
HNRNPL
FARSB
NARS
EPRS
LARS
***
*
PLA signal/cell
control
C9orf72

Supplementary Fig. 2 Antisense repeat RNA interaction with various proteins was tested in C9orf72 mutation-positive cells compared to control cells. a) Interactions of antisense repeat RNA with TAOK1, HNRNPL, HNRNPK, and FARSB, as revealed by the RNA-protein proximity ligation assay (PLA). In all three examples, the images above and below display C9orf72 and control fibroblasts, respectively. The PLA signals per cell for antisense repeat RNA interactions are 1.46 ± 0.38 (controls) and 5.44 ± 0.11 (C9orf72 fibroblasts) for TAOK1; 0.78 ± 0.15 (controls) and 3.57 ± 0.36 (C9orf72 fibroblasts) for HNRNPL; 8.17 ± 0.51 (controls) and 11.98 ± 1.03 (C9orf72 fibroblasts) for HNRNPK. The PLA signal did not increase for interactions between antisense repeat RNA and FARSB. b) Antisense repeat RNA PLA interactions with NARS, LARS, and EPRS in C9orf72 mutation-positive fibroblasts were as follows: 15.39 ± 1.13 (controls) and 18.67 ± 0.70 (C9orf72 fibroblasts) for NARS; 17.66 ± 0.67 (controls) and 19.05 ± 2.64 (C9orf72 fibroblasts) for LARS; 6.20 ± 0.83 (controls) and 7.29 ± 0.28 (C9orf72 fibroblasts) for EPRS. c) Antisense repeat RNA PLA interactions with NARS, LARS, and EPRS in C9orf72 mutation-positive lymphoblastoid cell lines are as follows: 4.60 ± 0.07 (controls) and 5.47 ± 0.44 (C9orf72 lymphoblastoid cell lines) for NARS; 5.27 ± 0.30 (controls) and 4.75 ± 0.36 (C9orf72 lymphoblastoid cell lines) for LARS; 6.46 ± 0.46 (controls) and 5.18 ± 0.65 (C9orf72 lymphoblastoid cell lines) for EPRS. d) Antisense repeat RNA PLA interactions with NARS, LARS, and EPRS in C9orf72 mutation-positive iPSCs are as follows: 0.89 ± 0.10 (controls) and 0.78 ± 0.04 (C9orf72 iPSCs) for NARS; 0.96 ± 0.07 (controls) and 1.01 ± 0.12 (C9orf72 iPSCs) for LARS; 0.66 ± 0.07 (controls) and 0.59 ± 0.13 (C9orf72 iPSCs) for EPRS. Scale bars: 10 µm. Graphs present mean values ± s.e.m. with statistical significance labeled as: * $p<0.05$, *** $p<0.001$. Source data are provided as a Source data file.

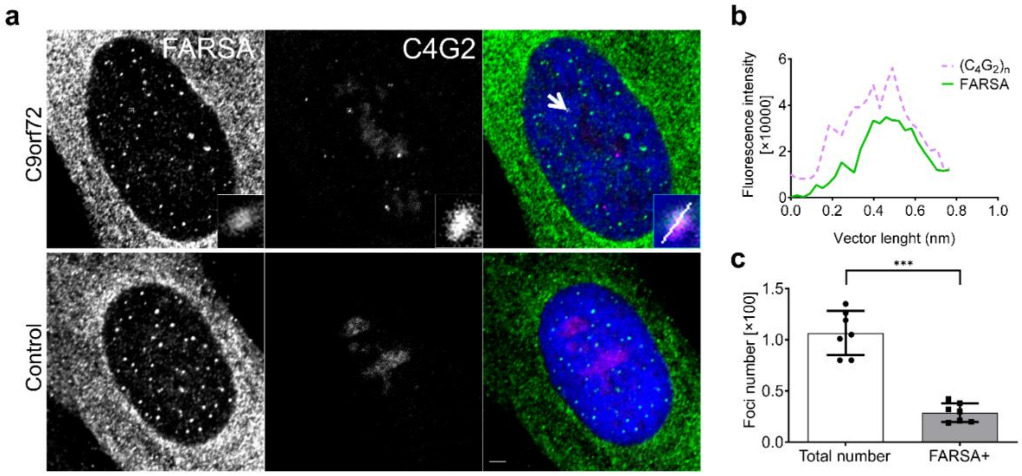

Supplementary Fig. 3 Nuclear antisense RNA foci co-localize with FARSA in C9orf72 patient-derived fibroblasts. Co-localization was detected with RNA fluorescent in situ hybridization in combination with immunofluorescent staining. Out of the total number of nuclear RNA foci, 27.5 ± 0.03% of foci co-localized with FARSA. Scale bars: 2.5 µm. Graphs present mean values ± s.e.m with statistical significance labeled as *** $p<0.001$. Source data are provided as a Source data file.

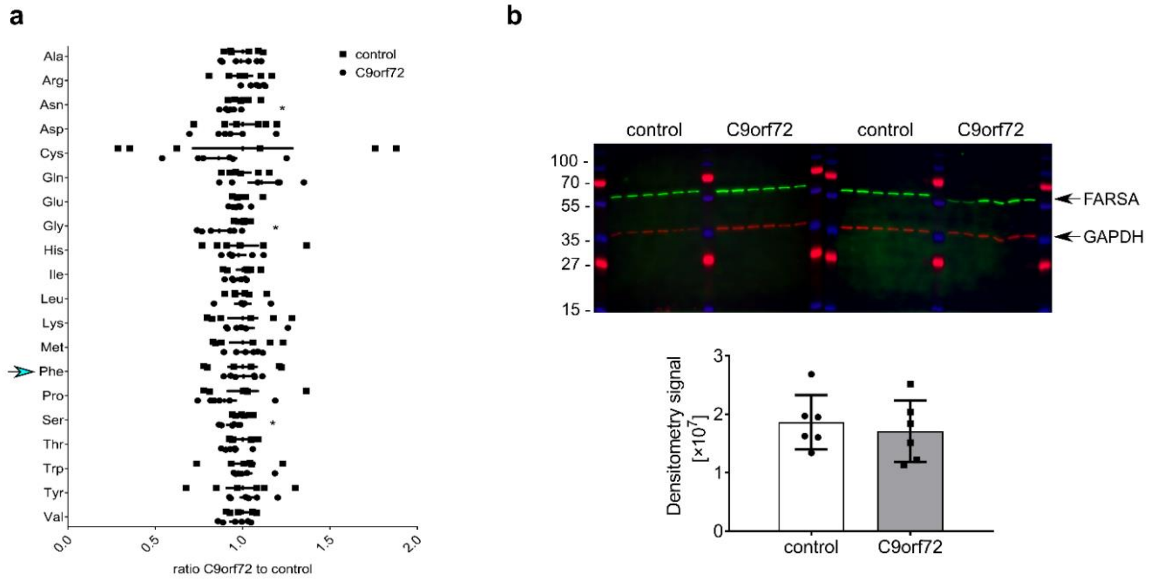

Supplementary Fig. 4 The concentration of free Phe and expression levels of FARSA are similar in control and C9orf72 cells. a) The concentrations of Phe (marked with an arrow) do not significantly differ between C9orf72 and control lymphoblastoid cell lines. The concentrations of total amino acids were used for normalization. Graphs present mean values ± s.e.m. with statistical significance labeled as: * $p<0.05$. b) FARSA expression levels are not significantly different between control and C9orf72 lymphoblastoid cell lines. Six control and six C9orf72 lines were used in technical duplicates. The graph presents mean value ± s.e.m. Source data are provided as a Source data file.

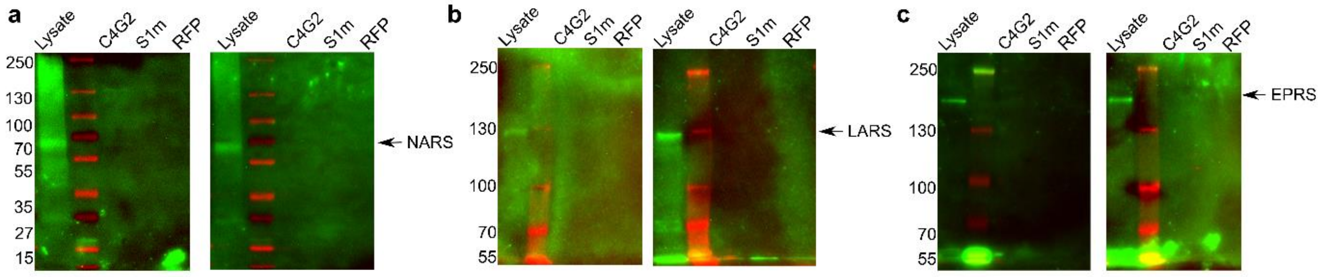

Supplementary Fig. 5 C4G2 RNA constructs do not bind different tRNA synthetases in the RNA pull-down assay. Western blots of eluates from RNA pull-down assays performed on human brain lysates revealed that C4G2 RNA repeats do not bind the following proteins: a) Asn-tRNA synthetase (NARS), b) Leu-tRNA synthetase (LARS) and c) Glu-Pro-tRNA synthetase (EPRS). Source data are provided as a Source data file.

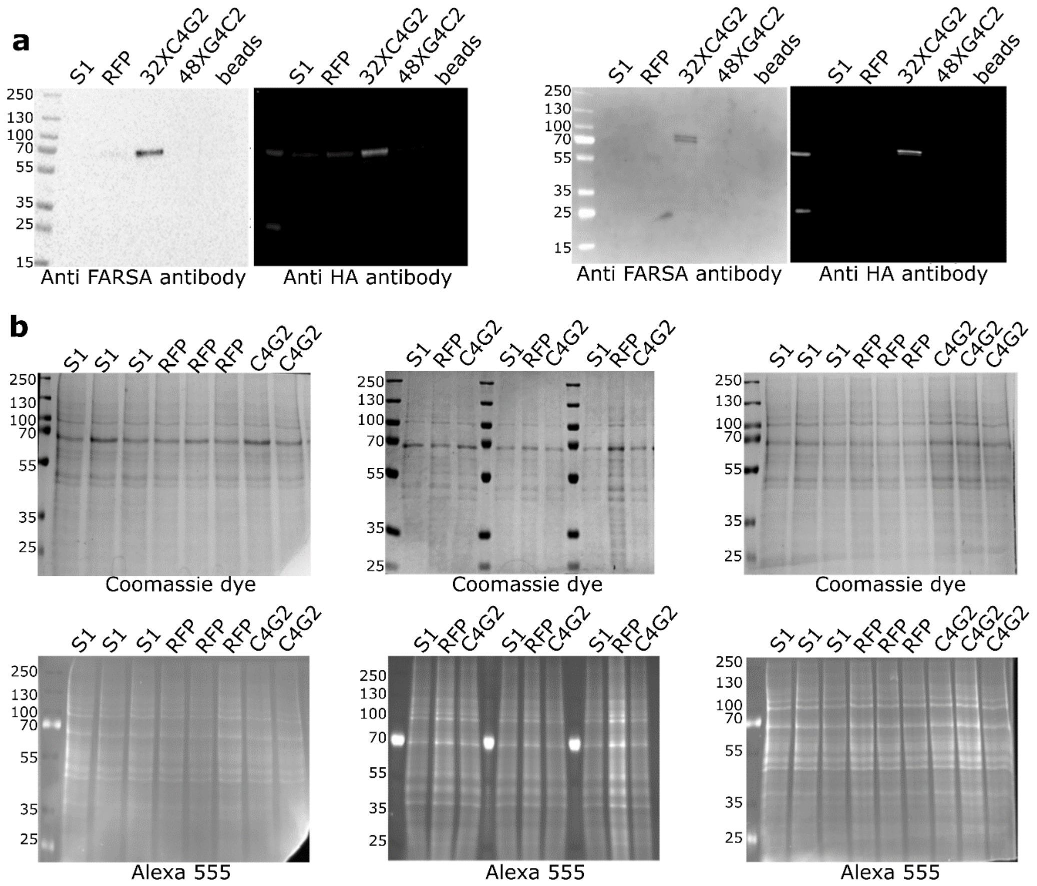

Supplementary Fig. 6 a) Mutated FARSA used in click chemistry experiment binds 32x C4G2 RNA constructs in RNA pull-down experiments on HEK293T cells. Images represent two independent repeats. b) Gels from additional click chemistry experiments are presented here. Source data are provided as a Source data file.

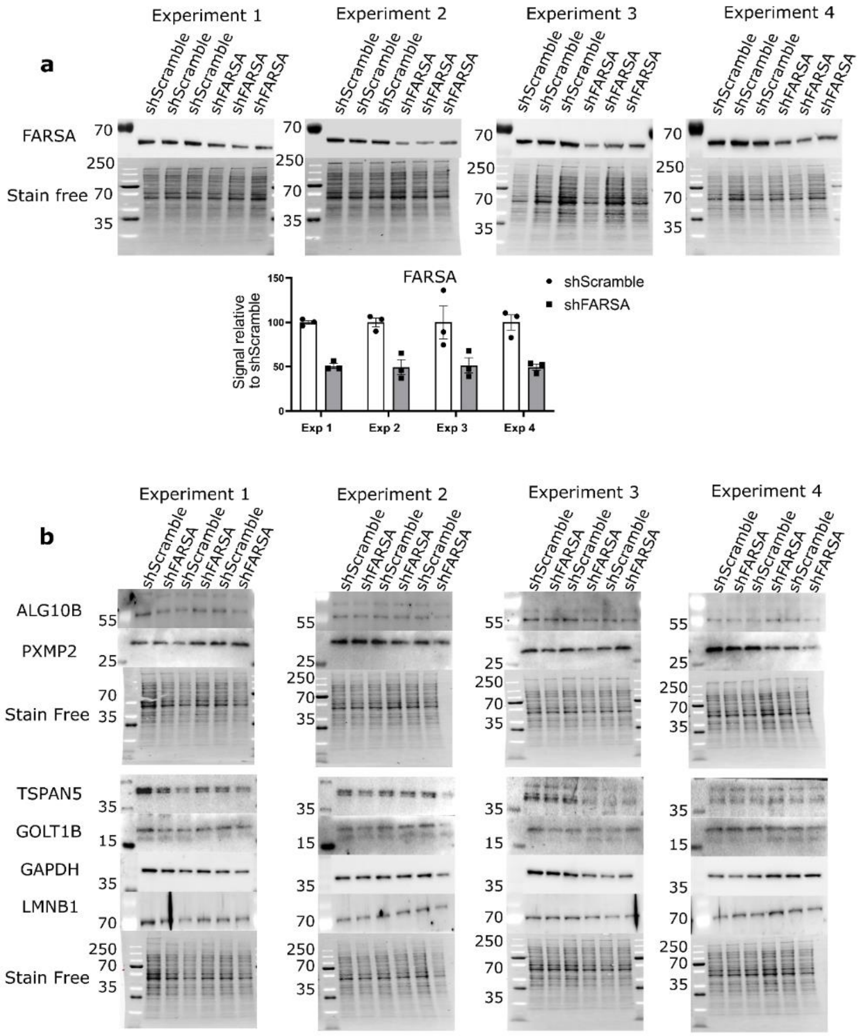

Supplementary Fig. 7 Expression of Phe-rich proteins is decreased in FARSA knockdown HEK293 cells. a) Expression of FARSA protein in HEK293 cells three days post lentivirus transduction in four independent repeats presented side by side. FARSA expression levels are presented relative to shScramble average (100 %) and are as follows: 50.7 ± 2.8 % (Ex1), 49.5 ± 8.3 % (Ex2), 43.2 ± 4.1 % (Ex3), 49.4 ± 3.6 % (Ex4). Graphs present mean values ± s.e.m. b) Western blots of four experiments evaluating Phe-rich protein expression in FARSA knockdown HEK293 cells. Stain free images represent loading comparison for all proteins above the stain free image and are vertically compressed for design reasons. Source data are provided as a Source data file.

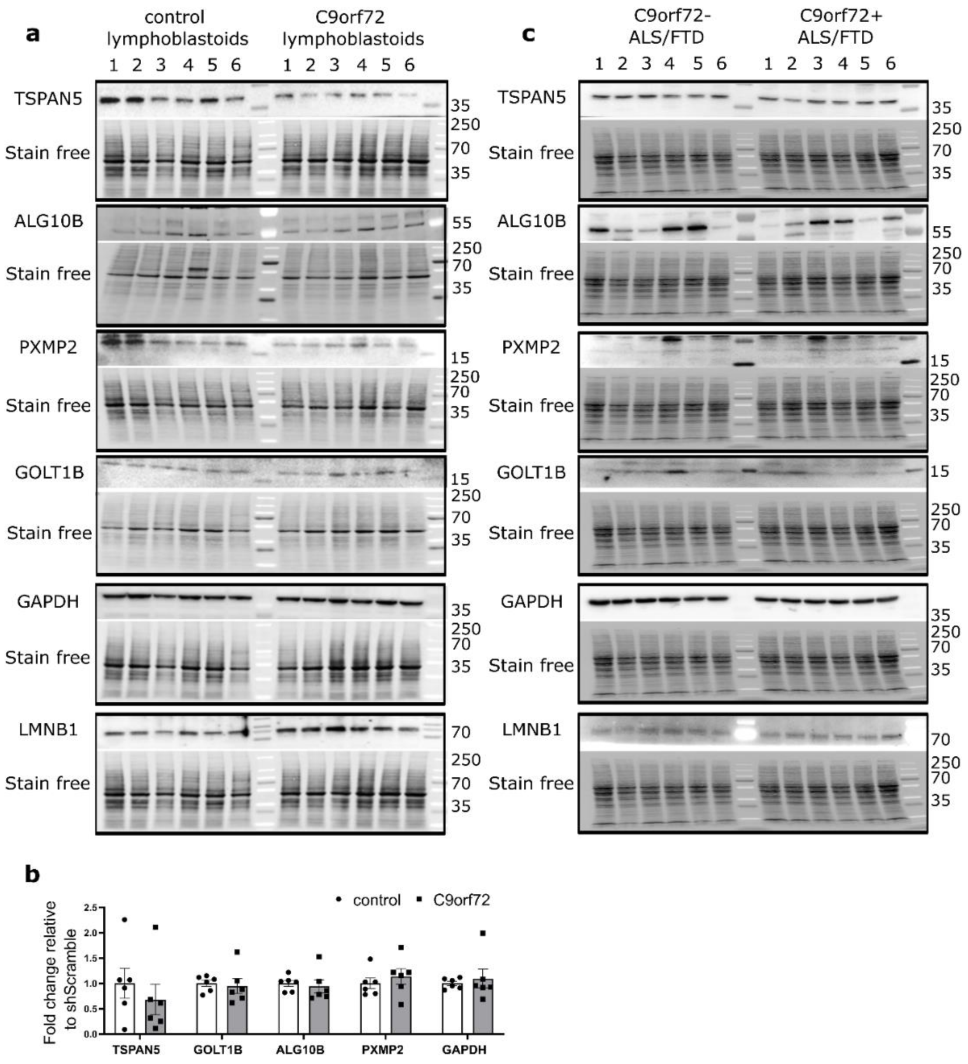

Supplementary Fig. 8 Expression of Phe-rich proteins is decreased in C9orf72-patient derived lymphoblastoid cell lines and post-mortem cerebellum tissue from C9orf72 ALS/FTD patients. a) Western blots from second technical repeat showing decreased expression of chosen Phe-rich proteins in 6 C9orf72-patient derived lymphoblastoid cell lines compared to 6 controls. b) qPCR analysis of Phe-rich protein mRNA transcripts revealed no significant changes in their transcription between C9orf72-patient derived lymphoblastoid cell lines and controls. Graphs present mean values normalized to *Actb* and relative to control average ± s.e.m. c) Additional western blots from RIPA lysates extracted from frozen cerebellar gray matter of 6 ALS/FTD cases with a C9orf72 mutation (C9+ ALS/FTD) and 6 controls (ALS/FTD cases without C9orf72 mutation, C9- ALS/FTD) showing decreased expression of ALG10B and TSPAN5 in C9orf72 cases. Stain free images represent loading comparison for all proteins above the stain free image and are vertically compressed for design reasons. Source data are provided as a Source data file.

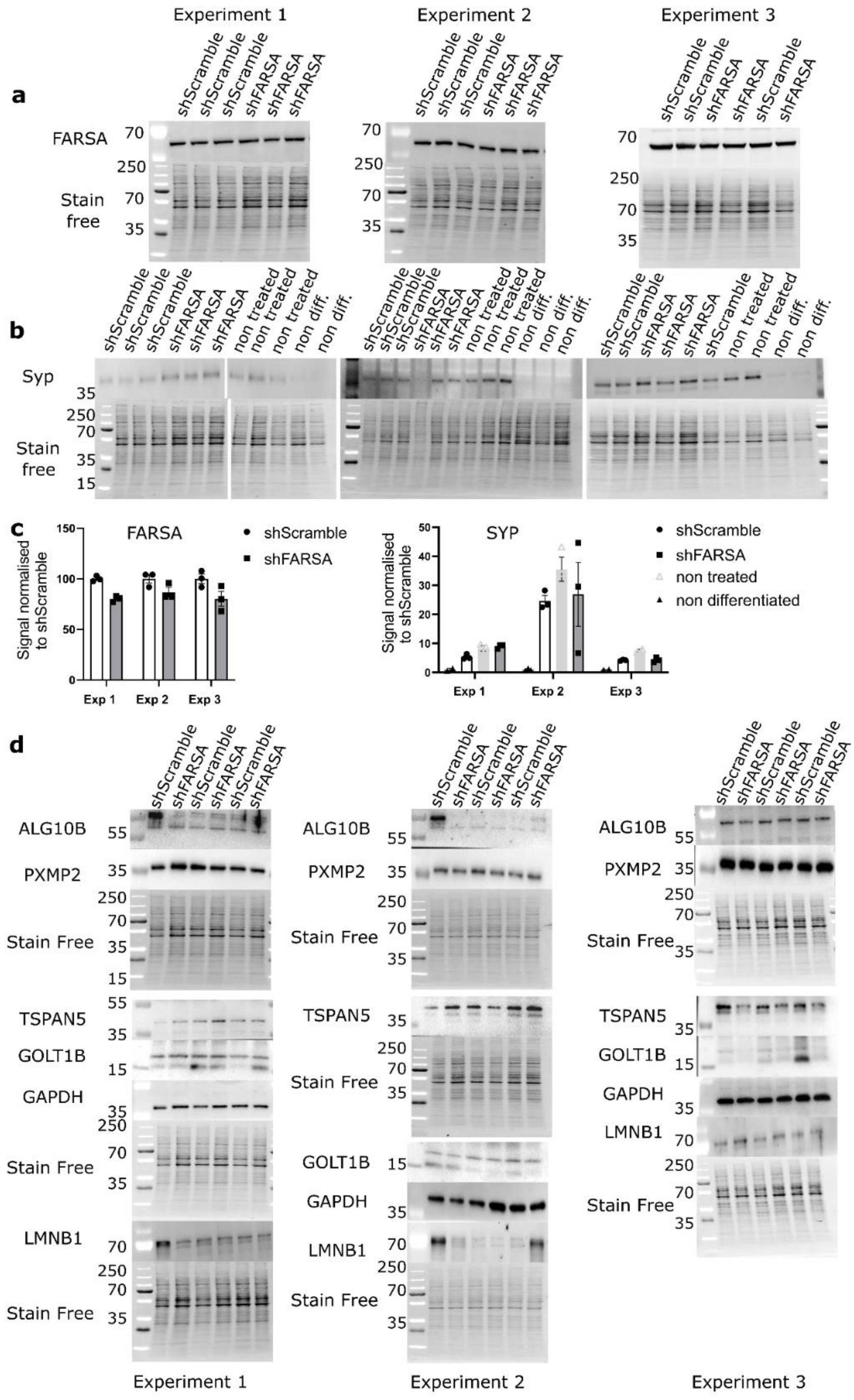

Supplementary Fig. 9 Expression of Phe-rich proteins is decreased in differentiated FARSA knockdown NSC-34 cells. a) Expression of FARSA protein in differentiated NSC-34 cells three days post lentivirus transduction for three independent experiments presented side by side. b) Expression of neuronal marker Synaptophysin (SYP) for all three experiments in differentiated and non-differentiated (non-diff.) NSC-34 cells. Non-treated cells are differentiated cells not exposed to lentiviruses. c)

Quantification of FARSA and SYP expression from (a) and (b). Values for FARSA expression relative to shScramble average (100 %) are as follows: 80.2 ± 1.7 % (Ex1), 82.2 ± 0.14 % (Ex2), 73.9 ± 5.9 % (Ex3). SYP expression relative to non-differentiated average is as follows: Ex1 non-differentiated 1 ± 0.5, non-treated 8.3 ± 0.9, shScramble 5.4 ± 0.5, shFARSA 9.0 ± 0.4; Ex2 non-differentiated 1 ± 0.2, non-treated 35.5 ± 4.2, shScramble 24.7 ± 1.8, shFARSA 26.9 ± 11.0; Ex3 non-differentiated 1 ± 0.01, non-treated 7.5 ± 0.5, shScramble 4.2 ± 0.3, shFARSA 4.4 ± 0.5. Graphs present mean values ± s.e.m. d) Western blots of three independent experiments showing reduced expression of ALG10B and TSPAN5 in FARSA knockdown differentiated NSC-34 cells. Stain free images represent loading comparison for all proteins above the stain free image and are vertically compressed for design reasons. Source data are provided as a Source data file.

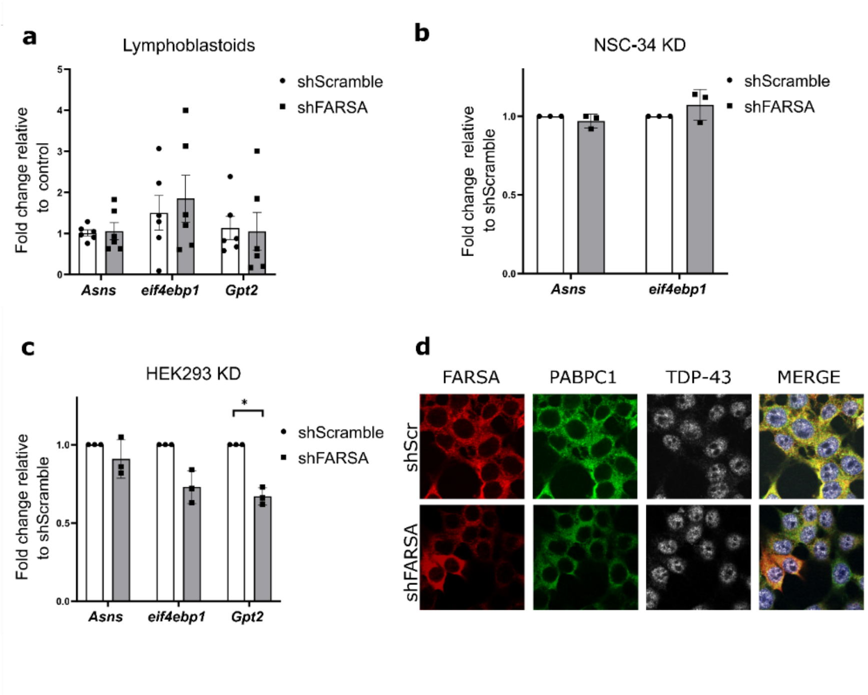

Supplementary Fig. 10 FARSA dysfunction does not induce stress response in C9orf72-patient derived lymphoblastoid cells, FARSA knockdown HEK293 and motor neuron-like NSC-34 cells. No increase in transcripts of stress response markers *Asns, Gpt2* and *eif4ebp1* was revealed by qPCR in a) C9orf72-mutation positive lymphoblastoid cell lines, b) differentiated FARSA knockdown NSC-34 and c) FARSA knockdown HEK293 cells. Data is normalised to *Actb* transcripts and relative to control lymphoblastoid cell lines or shScramble average. d) IF staining of FARSA knockdown HEK293 cells stained for FARSA (red), stress granule marker PABPC1 (green) and TDP-43 (white) shows no stress

response or TDP-43 aggregation. Graphs present mean values ± s.e.m. with statistical significance labeled as * $p<0.05$, ** $p<0.01$, *** $p<0.001$. Source data are provided as a Source data file.

Supplementary Tables:

Supplementary Table 1 Identified protein interactors of antisense repeats in cytoplasm and nuclear fraction from mass spectrometry analysis on mouse brain tissue. Protein candidates for further analysis were selected according to the cut-off criteria of a spectral count of > 20 and fold-change of > 3.

Nuclear fraction

Gel band	Protein name	Accession	Gene	MW (kDa)	C4G2	S1m	Fold change
1	Cytoplasmic FMR1-interacting protein 2	CYFP2_MOUSE	Cyfip2	146	41	8	5.13
	Contactin-1	CNTN1_MOUSE	Cntn1	113	54	17	3.18
	Serine/threonine-protein kinase TAO1	TAOK1_MOUSE	Taok1	116	73	4	18.25
	Serine/threonine-protein kinase TAO2	TAOK2_MOUSE	Taok2	139	54	14	3.86
2	Phenylalanine--tRNA ligase beta subunit	SYFB_MOUSE	Farsb	66	28	/	
	Heterogeneous nuclear ribonucleoprotein L	HNRPL_MOUSE	Hnrnpl	64	85	20	4.25
3	2',3'-cyclic-nucleotide 3'-phosphodiesterase	CN37_MOUSE	Cnp	47	76	9	8.44
	Flotillin-2	FLOT2_MOUSE	Flot2	47	19	4	4.75
	Flotillin-1	FLOT1_MOUSE	Flot1	48	20	/	
4	Nucleophosmin	NPM_MOUSE	Npm1	33	22	/	
	Eukaryotic translation initiation factor 2 subunit 1	IF2A_MOUSE	Eif2s1	36	13	/	

Cytoplasmic fraction

Gel band	Protein name	Accession	Gene	MW (kDa)	C4G2	S1m	Fold change
1	Phenylalanine--tRNA ligase beta subunit	SYFB_MOUSE	Farsb	66	159	21	7.57
2	Serine--tRNA ligase, cytoplasmic	SYSC_MOUSE	Sars	58	77	11	7.00
3	Phenylalanine--tRNA ligase alpha subunit	SYFA_MOUSE	Farsa	58	100	27	3.70

Supplementary Table 2 Detailed information about fibroblasts C9orf72 patient and control cell lines. F, female; M, male; N/A, not available

Cell line	Age	Sex	Disease type	Disease duration
C9orf72 fibroblasts-1	57	M	ALS-Leg	>4 years
C9orf72 fibroblasts-2	61	M	ALS-Bulbar	3.5 years
C9orf72 fibroblasts-3	42	F	ALS-Leg	N/A
Control fibroblasts-1	34	M	Control	N/A
Control fibroblasts-2	34	M	Control	N/A
Control fibroblasts-3	48	F	Control	N/A

Supplementary Table 3 Detailed information about lymphoblastoid C9orf72 patient and control cell lines. F, female; M, male

	Sex	Pathological Diagnosis
Human lymphoblastoid cell line, C9+	F	ALS
Human lymphoblastoid cell line, C9+	F	ALS
Human lymphoblastoid cell line, C9+	M	ALS
Human lymphoblastoid cell line, C9+	M	ALS
Human lymphoblastoid cell line, C9+	M	ALS
Human lymphoblastoid cell line, C9+	F	ALS
Human lymphoblastoid cell line, control	M	Non-affected parent of a child with LEUCODYSTROPHY
Human lymphoblastoid cell line, control	F	Non-affected parent of a child with LEUCODYSTROPHY
Human lymphoblastoid cell line, control	M	Non-affected parent of a child with LEUCODYSTROPHY
Human lymphoblastoid cell line, control	F	Non-affected parent of a child with LEUCODYSTROPHY
Human lymphoblastoid cell line, control	F	Non-affected parent of a child with LEUCODYSTROPHY
Human lymphoblastoid cell line, control	F	Non-affected parent of a child with LEUCODYSTROPHY

Supplementary Table 4 Detailed information about donors of cerebellum tissue donors. F, female; M, male; FTD, frontotemporal dementia; FTLD-TDP, frontotemporal lobar degeneration with TDP-43 pathology; ALS-TDP, amyotrophic lateral sclerosis with TDP-43 pathology; M, male; MND, motor neuron disease; N/A, not available

case #	C9orf72 mutation	Clinical diagnosis/phenotype	NP diagnosis	Sex	Age at death (years)	Disease duration (years)
1	no	MND	ALS-TDP	F	66	4
2	no	MND	ALS-TDP	M	60	13
3	no	MND	ALS-TDP	M	67	N/A
4	no	MND	ALS-TDP	F	72	3
5	no	MND	ALS-TDP	M	47	2
6	no	MND	ALS-TDP	F	67	5
7	no	MND	ALS-TDP	M	50	4
8	no	FTD	FTLD-TDP	F	63	5
9	no	FTD	FTLD-TDP	F	59	9
10	no	FTD/MND	FTLD/ALS-TDP	M	45	3
11	no	FTD/MND	FTLD/ALS-TDP	F	74	2
12	no	FTD/MND	FTLD/ALS-TDP	M	75	2
13	yes	MND	ALS-TDP	F	59	2
14	yes	MND	ALS-TDP	F	70	2
15	yes	MND	ALS-TDP	F	59	1
16	yes	MND	ALS-TDP	M	66	7
17	yes	FTD	FTLD-TDP	M	74	18
18	yes	FTD	FTLD-TDP	F	66	7
19	yes	FTD	FTLD-TDP	F	52	4
20	yes	FTD/MND	FTLD/ALS-TDP	M	55	3
21	yes	FTD/MND	FTLD/ALS-TDP	F	69	8
22	yes	FTD/MND	FTLD/ALS-TDP	F	61	3
23	yes	FTD/MND	FTLD/ALS-TDP	M	66	1
24	yes	FTD/MND	FTLD/ALS-TDP	M	61	4

Supplementary Table 5 Qpcr primers for observation of stress response activation.

ASNS human Fwd[1]	TGGTTGGTCCTCGCAGGCAT
ASNS human Rev[1]	CGCTTATACCGACCTGGCTCCT
GPT2 human Fwd[2]	ATGGCACTATGCACCTACCC
GPT2 human Rev[2]	CGGGCACGTTTCTTAGCATC
eif4EBP1 human Fwd[3]	AGCCCTTCCAGTGATGAGC
eif4EBP1 human Rev[3]	TGTCCATCTCAAACTGTGACTCTT
Asns mouse Fwd[4]	GGA TTG GCT GCC TTT TAT CA
Asns mouse Rev[4]	AAC TTG GGC CTC CTT GAG TT
eif4ebp1 mouse Fwd[5]	TGTCGGAACTCACCTGTG
eif4ebp1 mouse Rev[5]	GTCCCTTAAATGTCCATCTCAAA
Actb mouse Fwd[6]	CTAAGGCCAACCGTGAAAAGAT
Actb mouse Rev[6]	CACAGCCTGGATGGCTACGT

References:

1. Chen, H., Pan, Y. X., Dudenhausen, E. E. & Kilberg, M. S. Amino Acid Deprivation Induces the Transcription Rate of the Human Asparagine Synthetase Gene through a Timed Program of Expression and Promoter Binding of Nutrient-responsive Basic Region/Leucine Zipper Transcription Factors as Well as Localized Histone Acetylation. *J. Biol. Chem.* **279**, 50829–50839 (2004).

2. Zhang, B., Chen, Y., Bao, L. & Luo, W. GPT2 Is Induced by Hypoxia-Inducible Factor (HIF)-2 and Promotes Glioblastoma Growth. *Cells* **11**, (2022).

3. Hauffe, L. *et al.* Eukaryotic translation initiation factor 4E binding protein 1 (EIF4EBP1) expression in glioblastoma is driven by ETS1- and MYBL2-dependent transcriptional activation. *Cell Death Discov. 2022 81* **8**, 1–11 (2022).

4. Iida, S. *et al.* Reduced Plasma Glucose by Asparagine Synthetase Knockdown in the Mouse Liver. *Biol. Pharm. Bull.* **36**, 2009–2011 (2013).

5. Chang, S. H. *et al.* Aerosol delivery of eukaryotic translation initiation factor 4E-binding protein 1 effectively suppresses lung tumorigenesis in K-rasLA1 mice. *Cancer Gene Ther. 2013 206* **20**, 331–335 (2013).

6. Areal, C. C., Cao, R., Sonenberg, N. & Mongrain, V. Wakefulness/sleep architecture and electroencephalographic activity in mice lacking the translational repressor 4E-BP1 or 4E-BP2. *Sleep* **43**, 1–12 (2020).
