# Supplementary material for: Phenylalanine-tRNA aminoacylation is compromised by ALS/FTD-associated C9orf72 C4G2 repeat RNA
Source: Nat Commun. 2023 Sep 16;14:5764. doi: 10.1038/s41467-023-41511-3 (PMC10505166; doi:10.1038/s41467-023-41511-3)
Supplement: Supplementary file 3 — Description of Additional Supplementary Files [file 41467_2023_41511_MOESM3_ESM.pdf]

Description of Additional Supplementary Files

File Name: Supplementary Data 1

Description: **Identified proteins from mass spectrometry analysis of C9orf72 mutation positive lymphoblasts and control RIPA fraction.** Table represents all protein detected, proteins used for quantification and analysis of ratio of downregulated and upregulated proteins in relation to Phe content. The parameters including statistical tests were left at default in Proteome discoverer software if not otherwise specified in the Methods section
