# Supplementary material for: Phenylalanine-tRNA aminoacylation is compromised by ALS/FTD-associated C9orf72 C4G2 repeat RNA
Source: Nat Commun. 2023 Sep 16;14:5764. doi: 10.1038/s41467-023-41511-3 (PMC10505166; doi:10.1038/s41467-023-41511-3)
Supplement: Supplementary file 5 — Reporting Summary [file 41467_2023_41511_MOESM5_ESM.pdf]

nature portfolio

Corresponding author(s): Boris Rogelj
Last updated by author(s): Aug 17, 2023

Reporting Summary

Nature Portfolio wishes to improve the reproducibility of the work that we publish. This form provides structure for consistency and transparency in reporting. For further information on Nature Portfolio policies, see our Editorial Policies and the Editorial Policy Checklist.

Statistics

For all statistical analyses, confirm that the following items are present in the figure legend, table legend, main text, or Methods section.

n/a	Confirmed	
☐	☒	The exact sample size (*n*) for each experimental group/condition, given as a discrete number and unit of measurement
☐	☒	A statement on whether measurements were taken from distinct samples or whether the same sample was measured repeatedly
☐	☒	The statistical test(s) used AND whether they are one- or two-sided<br>*Only common tests should be described solely by name; describe more complex techniques in the Methods section.*
☒	☐	A description of all covariates tested
☐	☒	A description of any assumptions or corrections, such as tests of normality and adjustment for multiple comparisons
☐	☒	A full description of the statistical parameters including central tendency (e.g. means) or other basic estimates (e.g. regression coefficient) AND variation (e.g. standard deviation) or associated estimates of uncertainty (e.g. confidence intervals)
☐	☒	For null hypothesis testing, the test statistic (e.g. *F*, *t*, *r*) with confidence intervals, effect sizes, degrees of freedom and *P* value noted<br>*Give P values as exact values whenever suitable.*
☒	☐	For Bayesian analysis, information on the choice of priors and Markov chain Monte Carlo settings
☒	☐	For hierarchical and complex designs, identification of the appropriate level for tests and full reporting of outcomes
☒	☐	Estimates of effect sizes (e.g. Cohen's *d*, Pearson's *r*), indicating how they were calculated

Our web collection on statistics for biologists contains articles on many of the points above.

Software and code

Policy information about availability of computer code

Data collection	ImageLab 6.1 software (Bio-Rad, Hercules, California, USA) was used for western blot image capture. ZEN black 3.0 software was used for confocal image capture.
Data analysis	Mass spectrometry data on mouse brain samples were analyzed with Proteome Discoverer (version 2.1, Thermo Fisher Scientific) against UniProt/SwissProt mouse database (version 2017_01, 16,844 protein entries) using Mascot (version 2.6.0, Matrix Sciences).<br>Mass spectrometry data from lymphoblastoid cell lines were searched against human protein database (UniProt/SwissProt Release 2022_01, 20376 protein entries) combined with FBS contamination database (249 protein entries) using Mascot algorithm (version 2.6.0, Matrix Science) and Proteome Discoverer (version 2.4.1.15, Thermo Fisher Scientific)<br>ImageJ was used for immunofluorescence and proximity-ligaton assays analysis. ImageJ was used for click-chemistry analysis. ImageLab 6.1 software (Bio-Rad, Hercules, California, USA) was used for western blot analysis.<br>Amino acid analysis was done with Cliquid software (version 3.2.1., Sciex) in conjunction with Analyst software (version 1.6.2, Sciex).<br>Statistic was performed and graphs were designed in Excel 2010 or GrafpadPrism version 9.5.0.

For manuscripts utilizing custom algorithms or software that are central to the research but not yet described in published literature, software must be made available to editors and reviewers. We strongly encourage code deposition in a community repository (e.g. GitHub). See the Nature Portfolio guidelines for submitting code & software for further information.

Data

Policy information about availability of data

All manuscripts must include a data availability statement. This statement should provide the following information, where applicable:

- Accession codes, unique identifiers, or web links for publicly available datasets
- A description of any restrictions on data availability
- For clinical datasets or third party data, please ensure that the statement adheres to our policy

Data are available via ProteomeXchange with identifier PXD024527 and PXD042474.
Project accession: PXD024527 and PXD042474

Human research participants

Policy information about studies involving human research participants and Sex and Gender in Research.

Reporting on sex and gender	Research does not include human participants.
Population characteristics	N/A
Recruitment	N/A
Ethics oversight	N/A

Note that full information on the approval of the study protocol must also be provided in the manuscript.

Field-specific reporting

Please select the one below that is the best fit for your research. If you are not sure, read the appropriate sections before making your selection.

☒ Life sciences ☐ Behavioural & social sciences ☐ Ecological, evolutionary & environmental sciences

For a reference copy of the document with all sections, see nature.com/documents/nr-reporting-summary-flat.pdf

Life sciences study design

All studies must disclose on these points even when the disclosure is negative.

Sample size	No sample-size calculation was performed; sample sizes were chosen based on the availability of patient-derived cell lines with the goal of including at least three different cell lines for each experiment.
Data exclusions	Data of ineficient knockdown experiments were excluded. Also if they were unquantifiable because of unclean signal on WB. All is recorded in source data and uncropped WB file.
Replication	Reproducibility of the experiments was tested in biological and technical replicates. The data presented in the manuscript was reproducible in the replication experiments. Initial RNA-pull down experiments (Figure 1) were done once and the following validation experiments were done in at least 3 independent experiments or on at least 4 independent biological samples for each condition. The exact number of performed experiments is stated in the manuscript and Figure legends. All data is persented in Source data file.
Randomization	The samples were not randomized. There were two groups analyzed in the manuscript: control group (cells derived from healthy individuals) and disease group (cells derived from patients with C9orf72 mutation).
Blinding	The blinding of investigators to group allocation was not possible as individual investigator was responsible for the whole process from cell maintenance, sample collection and analysis. In our opinion, blinding of the investigators was not relevant for this study as this was an exploratory research to find proteins binding to the selected RNA and investigators did not hold prior bias to expected results. Negative results are also included in the manuscript as proof of this concept. Furthermore, findings indicating same functional consequences were result of different types of experiments performed by different investigators on different locations.

Reporting for specific materials, systems and methods

We require information from authors about some types of materials, experimental systems and methods used in many studies. Here, indicate whether each material, system or method listed is relevant to your study. If you are not sure if a list item applies to your research, read the appropriate section before selecting a response.

Materials & experimental systems

n/a	Involved in the study
	☒ Antibodies
	☒ Eukaryotic cell lines
☒	Palaeontology and archaeology
	☒ Animals and other organisms
☒	Clinical data
☒	Dual use research of concern

Methods

n/a	Involved in the study
☒	ChIP-seq
☒	Flow cytometry
☒	MRI-based neuroimaging

Antibodies

Antibodies used

FARSA Polyclonal antibody, 18121-1-AP, lot: 00009936, Proteintech, Rosemont, Illinois, USA
FARSB Polyclonal antibody, 16341-1-AP, lot: 00007840 Proteintech, Rosemont, Illinois, USA
TAOK1 Polyclonal antibody, 26250-1-AP, lot: 00041178, Proteintech, Rosemont, Illinois, USA
CNPase Polyclonal antibody, 13427-1-AP, lot: 00043126, Proteintech, Rosemont, Illinois, USA
CYFIP1/2 Polyclonal antibody, 16011-1-AP, lot: 00007263, Proteintech, Rosemont, Illinois, USA
GAPDH Monoclonal antibody, 60004-1-Ig, lot: 10013030, Proteintech, Rosemont, Illinois, USA
GAPDH Polyclonal antibody, 10494-1-AP, lot: 00087635, Proteintech, Rosemont, Illinois, USA
TSPAN5 Polyclonal antibody, 12122-1-AP, lot: 00002931, Proteintech, Rosemont, Illinois, USA
PXMP2 Polyclonal antibody, 24801-1-AP, lot: 00021277, Proteintech, Rosemont, Illinois, USA
LARS Polyclonal antibody, 21146-1-AP, lot: 00014501, Proteintech, Rosemont, Illinois, USA
NARS Polyclonal antibody, 14882-1-AP, lot: 00042769, Proteintech, Rosemont, Illinois, USA
EPRS Polyclonal antibody, 25307-1-AP, lot: 00023786, Proteintech, Rosemont, Illinois, USA
HA Tag Monoclonal antibody, 66006-2-Ig, lot: 10011878, Proteintech, Rosemont, Illinois, USA
hnRNP L Antibody, sc-32317, lot: 4D11, Santa Cruz Biotechnology, Dallas, Texas, USA
hnRNP K Antibody, sc-28380, lot:D-6, Santa Cruz Biotechnology, Dallas, Texas, USA
B23/Nucleophosmin Antibody, sc-56622, lot: FC82291, Santa Cruz Biotechnology, Dallas, Texas, USA
Lamin B1 Antibody, sc20682, lot: H-90, Santa Cruz Biotechnology, Dallas, Texas, USA
Syp/Synaptophisin, sc-17750, lot: D0419, Santa Cruz Biotechnology, Dallas, Texas, USA
Histone H3 Antibody Polyclonal, NB500-171, Novus Biologicals, Centennial, Colorado, USA
ALG10b Antibody Polyclonal, NBP3-09560, lot: QC37618-41080, Novus Biologicals, Centennial, Colorado, USA
Anti-Biotin antibody [Hyb-8], ab201341, lot: GR3446252-1, Abcam, Cambridge, UK
TDP-43, ab80608, lot: GR83948-15, Abcam, Cambridge, UK
Anti-biotin (D5A7) Rabbit mAb. 5597S, lot: D5A7, Cell Signaling Technology, Danvers, Massachusetts, USA
GOLT1B , PA5-103499, lot: WI3392211 , Invitrogen, Waltham, Massachusetts, ZDA
Anti-GOLT1B antibody produced in rabbit polyclonal, HPA055909, Sigma Aldrich, St. Louis, Missouri, USA; WB 1:200).
PABPC1, P6246, lot: 015M4782V, Sigma Aldrich, St. Louis, Missouri, USA
Anti-mouse IgG (H+L), F(ab')2 Fragment (Alexa Fluor® 488 Conjugate), b4408, lot: 4408S, Cell Signaling Technology, Danvers, Massachusetts, USA
Donkey anti-Mouse IgG (H+L) Highly Cross-Adsorbed Secondary Antibody, Alexa Fluor™ 488, A21202, lot: 2253917, Invitrogen, Waltham, Massachusetts, ZDA
Donkey anti-Rabbit IgG (H+L) Highly Cross-Adsorbed Secondary Antibody, Alexa Fluor™ 555, A31572, lot: 2180682, Invitrogen, Waltham, Massachusetts, ZDA
Donkey anti-Goat IgG (H+L) Cross-Adsorbed Secondary Antibody, Alexa Fluor™ 647, A21447, lot: 2045332, Invitrogen, Waltham, Massachusetts, ZDA
StarBright™ Blue 520 Goat Anti-Mouse IgG, #12005866, Bio-Rad, Hercules, California, USA
StarBright™ Blue 520 Goat Anti-Rabbit IgG, #12005869, lot: 64440259, Bio-Rad, Hercules, California, USA
StarBright Blue 700 Goat Anti-Mouse IgG, #12004158, lot: 64371260, Bio-Rad, Hercules, California, USA
StarBright Blue 700 Goat Anti-Rabbit IgG, #12004161, Bio-Rad, Hercules, California, USA
Peroxidase AffiniPure Goat Anti-Rabbit IgG (H+L),111-035-045, lot: 162297, Jackson ImmunoResearch, West Grove, Pennsylvania, USA
Peroxidase AffiniPure Goat Anti-Mouse IgG (H+L), 115-035-044, lot: 159331, Jackson ImmunoResearch, West Grove, Pennsylvania, USA
Normal Rabbit IgG, 12-370, lot: 3493998, Millipore, Burlington, Massachusetts, USA

Validation

-FARSA Polyclonal antibody, 18121-1-AP, Proteintech – reactivity Hu, Mu, Rt; validated for use in Western Blotting, Immunoprecipitation, Immunofluorescence, Immunohistochemistry (validation statement and publications listed on the manufacturer`s website), Proximity Ligation Assay (data provided in the manuscript)
-FARSB Polyclonal antibody, 16341-1-AP, Proteintech – reactivity Hu, Mu, Rt; validated for use in Western Blotting, Immunoprecipitation, Immunofluorescence, Immunohistochemistry (validation statement and publications listed on the manufacturer`s website), Proximity Ligation Assay (data provided in the manuscript)
-TAOK1 Polyclonal antibody, 26250-1-AP, Proteintech – reactivity Hu, Mu, Mk; validated for use in Western Blotting, Immunofluorescence, Immunohistochemistry (validation statement and publications listed on the manufacturer`s website), Proximity Ligation Assay (data provided in the manuscript)
-CNPase Polyclonal antibody, 13427-1-AP, Proteintech – reactivity Hu, Mu, Rt; validated for use in Western Blotting, Immunoprecipitation, Immunofluorescence, Immunohistochemistry (validation statement and publications listed on the manufacturer`s website)
-CYFIP1/2 Polyclonal antibody, 16011-1-AP, Proteintech – reactivity Hu, Mu; validated for use in Western Blotting, Immunoprecipitation, Immunofluorescence, Immunohistochemistry (validation statement and publications listed on the manufacturer`s website)

-GAPDH Monoclonal antibody, 60004-1-Ig, Proteintech – reactivity Hu, Mu, Rt, Bv, At, Ce, Ca, Ch, Pl, Ye, Ze; validated for use in Western Blotting, Immunoprecipitation, Immunofluorescence, Immunohistochemistry (validation statement and publications listed on the manufacturer`s website)
-GAPDH Polyclonal antibody, 10494-1-AP, Proteintech – reactivity Hu, Mu, Rt, Po, At, Ce, Ca, Ch, Ha, Fi, Gt, Pl; validated for use in Western Blotting, Immunoprecipitation, Immunofluorescence, Immunohistochemistry (validation statement and publications listed on the manufacturer`s website)
-TSPAN5 Polyclonal antibody, 12122-1-AP, Proteintech – reactivity Mu, Rt, Hu, validated for use in Western Blotting (validation statement and publications listed on the manufacturer`s website)
-PXMP2 Polyclonal antibody, 24801-1-AP, Proteintech – reactivity Mu, Rt, Hu; validated for use in Western Blotting, Immunohistochemistry (validation statement and publications listed on the manufacturer`s website)
-LARS Polyclonal antibody, 21146-1-AP, Proteintech – reactivity Hu; validated for use in Western Blotting, Immunoprecipitation, Immunofluorescence, Immunohistochemistry, KD/KO validated (validation statement and publications listed on the manufacturer`s website), Proximity Ligation Assay (data provided in the manuscript)
-NARS Polyclonal antibody, 14882-1-AP, Proteintech – reactivity Hu; validated for use in Western Blotting, Immunoprecipitation, Immunohistochemistry (validation statement and publications listed on the manufacturer`s website), Proximity Ligation Assay (data provided in the manuscript)
-EPRS Polyclonal antibody, 25307-1-AP, Proteintech – reactivity Hu; validated for use in Western Blotting, Immunofluorescence; (validation statement and publications listed on the manufacturer`s website), Proximity Ligation Assay (data provided in the manuscript)
-HA Tag Monoclonal antibody, 66006-2-Ig – reactivity Mu, Hu, Po, Duck; validated for use in Western Blotting, Immunoprecipitation, Chromatin Immunoprecipitation, Immunofluorescence, ELISA (validation statement and publications listed on the manufacturer`s website)
-hnRNP L Antibody (4D11), sc-32317, Santa Cruz Biotechnology – reactivity Mu, Hu, Rt; validated for use in Western Blotting, Immunoprecipitation, Immunofluorescence, Immunohistochemistry (validation statement and publications listed on the manufacturer`s website)
-hnRNP K Antibody (D-6), sc-28380, Santa Cruz Biotechnology – reactivity Mu, Hu, Rt; validated for use in Western Blotting, Immunoprecipitation, Immunofluorescence, Immunohistochemistry, ELISA (validation statement and publications listed on the manufacturer`s website), Proximity Ligation Assay (data provided in the manuscript)
-B23/Nucleophosmin Antibody (FC82291), sc-56622, Santa Cruz Biotechnology, Dallas, Texas, USA - reactivity Mu, Rt, Hu, Bv, Ca; validated for use in Western Blotting, Immunoprecipitation, Immunofluorescence, Immunohistochemistry, ELISA (validation statement and publications listed on the manufacturer`s website)
-Lamin B1 Antibody (H-90), sc20682, Santa Cruz Biotechnology – validated for use in Western Blotting (publications listed on the manufacturer`s website), discontinued
-Histone H3 Antibody Polyclonal, NB500-171, Novus Biologicals – reactivity Hu, Mu, Rt, I, Xp, Ye, Ce, Ch, Dr, Pl; validated for use in Western Blotting, Chromatin Immunoprecipitation, Immunoblotting, Immunocytochemistry/Immunofluorescence, Immunohistochemistry (validation statement and publications listed on the manufacturer`s website)
-ALG10b Antibody Polyclonal, NBP3-09560, Novus Biologicals - reactivity Hu; validated for use in Western Blotting (validation statement on the manufacturer`s website)
-Anti-Biotin antibody [Hyb-8], ab201341, Abcam – species independent; validated for use in Flow Cytometry, In situ hybridization, Western Blotting, Immunocytochemistry, Immunofluorescence, Immunohistochemistry (validation statement on the manufacturer`s website)
-Anti-biotin (D5A7) Rabbit mAb. 5597S, Cell Signaling Technology - species independent; validated for use in Western Blotting & Elisa (validation statement on the manufacturer`s website), validated for use in Western Blotting, Immunohistochemistry & Proximity Ligation Assay (citations provided on the manufacturer`s website)
-Anti-GOLT1B antibody produced in rabbit polyclonal, PA5-103499, Invitrogen – reactivity Hu, Ms, Rat; Western Blotting (data provided in the manuscript)
-Anti-GOLT1B antibody produced in rabbit polyclonal, HPA055909, Sigma Aldrich – reactivity Hu; Western Blotting (data provided in the manuscript)
-Normal Rabbit IgG, 12-370, Millipore - validated for use in Immunoprecipitation & Western Blotting (validation statement on the manufacturer`s website)
-Anti Syp/Synaptophysin antibody produced in mouse monoclonal, sc-17750, SantaCruz Biotechnology- reactivity Hu, Ms, Rat; Western Blotting (data provided in the manuscript
- Anti TDP-43 antibody produced in goat polyclonal, ab80608, Abcam, reactivity- Hu; IF (data provided in the manuscript)
- Anti PABPC1 antibody produced in mouse monoclona, P6246, Sigma-Aldrich, reactivity - Hu, Ms, Rat; IF (data provided in the manuscript

Eukaryotic cell lines

Policy information about cell lines and Sex and Gender in Research

Cell line source(s)

HEK293T cells were obtained from ATTC (CRL-3216).
HEK293 cells were obtained from ATTC (CRL-1573).
NSC-34 cells were obtained from Cedarlane Laboratories (CLU140).
C9orf72 patient-derived (cell line CS52iALS-C9n6) and isogenic control (cell line CS52iALS-C9n6.ISOC3) induced pluripotent stem cells (iPSCs) were purchased from Cedars-Sinai Medical Center (LA, California; https://www.cedars-sinai.org/research/areas/biomanufacturing/ipsc.html).

C9orf72 patient-derived fibroblasts were a kind gift from Dr. Don W. Cleveland (Ludwig Institute for Cancer Research, La Jolla, California, USA) (Lagier-Tourenne, 2013). Control fibroblasts were obtained from skin biopsies of healthy volunteers and approved by the Ethical Committee (EK45022009) of the Technische Universität Dresden, Germany (Lojewski, 2014).

Cell line /Age/Sex/Disease type /Disease duration
C9orf72 fibroblasts-1/57/M/ALS-Leg/ >4 years
C9orf72 fibroblasts-2/61/M/ALS-Bulbar/ 3.5 years
C9orf72 fibroblasts-3/42/F/ALS-Leg/ N/A

Control fibroblasts-1/34 /M/Control/ N/A
Control fibroblasts-2/34 /M/ Control/ N/A
Control fibroblasts-3/48/F/Control/ N/A

C9orf72 patient-derived and control lymphoblastoid cell lines were prepared from patients segregating C9orf72 mutations as previously described (Daoud, 2012).

Cell line/Date of biopsy/Sex/Pathological Diagnosis
Human lymphoblastoid cell line, C9+; R0041494/9.27.1963/F/ALS
Human lymphoblastoid cell line, C9+; R0041495/12.31.1953/F/ALS
Human lymphoblastoid cell line, C9+; R0042873/5.26.1955/M/ALS
Human lymphoblastoid cell line, C9+; R0041487/12.7.1966/M/ALS
Human lymphoblastoid cell line, C9+; R0042890/7.13.1954/M/ALS
Human lymphoblastoid cell line, C9+; R0044182/2.9.1938/F/ALS
Human lymphoblastoid cell line, control; R0044939/7.30.1981/M/Non-affected parent of a child with LEUCODYSTROPHY
Human lymphoblastoid cell line, control; R0044940/4.29.1982/F/Non-affected parent of a child with LEUCODYSTROPHY
Human lymphoblastoid cell line, control; R0044625/5.10.1963/M/Non-affected parent of a child with LEUCODYSTROPHY
Human lymphoblastoid cell line, control; R0044713/8.14.1978/F/Non-affected parent of a child with LEUCODYSTROPHY
Human lymphoblastoid cell line, control; R0044922/3.18.1974/F/Non-affected parent of a child with LEUCODYSTROPHY
Human lymphoblastoid cell line, control; R0045439/4.4.1981/F/Non-affected parent of a child with LEUCODYSTROPHY

Authentication

Cell lines were not additionally authenticated.

Mycoplasma contamination

All cell lines tested negative for mycoplasma contamination

Commonly misidentified lines
(See ICLAC register)

no commonly misidentified cell lines were used in the study.

Animals and other research organisms

Policy information about studies involving animals; ARRIVE guidelines recommended for reporting animal research, and Sex and Gender in Research

Laboratory animals

Mus musculus, FVB/PyMT, age 2y, female

Wild animals

Study did not involve wild animals.

Reporting on sex

Sex was not considered in the study design. The study used tissue collected from mice only as a protein source material for immunoprecipitation experiment, therefore, no sex-based analyses were conducted.

Field-collected samples

Study did not involve samples collected from the field.

Ethics oversight

Mouse brains were obtained under approval of the Veterinary Administration of the Ministry of Agriculture and the Environment, Slovenia.

Note that full information on the approval of the study protocol must also be provided in the manuscript.
